# Addition of Epigallocatechin Gallate 400 mg to Sofosbuvir 400 mg + Daclatisvir 60 mg With or Without Ribavirin in Treatment of Patients with Chronic Hepatitis C Improves the Safety Profile: A Pilot Study

**DOI:** 10.1038/s41598-019-49973-6

**Published:** 2019-09-19

**Authors:** Gamal Shiha, Reham Soliman, Mohamed Elbasiony, Noureldien H. E. Darwish, Shaker A. Mousa

**Affiliations:** 1 Internal Medicine Department, Faculty of Medicine, Mansoura University, Mansoura, Egypt; 2 Egyptian Liver Research Institute and Hospital (ELRIAH), Mansoura, Egypt; 3 Tropical Medicine Department, Faculty of Medicine, Port Said University, Port Said, Egypt; 4 Clinical Pathology Department, Faculty of Medicine, Mansoura University, Mansoura, Egypt; 5 The Pharmaceutical Research Institute, Albany College of Pharmacy and Health Sciences, Rensselaer, NY USA; 6 Virothera Pharmaceuticals, Rensselaer, NY USA

**Keywords:** Inflammatory diseases, Hepatitis C

## Abstract

Emergence of new molecules acting directly on the hepatitic C virus (HCV) has improved treatment outcomes. However, there is a risk of selecting viral escape mutants, so a new combination is needed using different inhibitors that target different steps of the HCV infectious cycle. Novel single tablet formulations were developed: Dactavira, composed of sofosbuvir (SOF) 400 mg/daclatisvir (DCV) 60 mg/epigallocatechin gallate (EGCG) 400 mg without ribavirin (RBV); and Dactavira plus, which includes RBV 800 mg. A randomized, open-label study was carried out on treatment-naïve non-cirrhotic (Group A, n = 50) and treatment-naïve cirrhotic (Group B, n = 22) patients with genotype 4 HCV infection. Group A was randomly assigned to receive a single daily fixed-dose (Dactavira, n = 25) or the standard of care [SOF 400 mg/DCV 60 mg] (n = 25) daily for 12 weeks. Group B was randomly assigned to receive a single daily fixed-dose (Dactavira plus, n = 11) or the standard of care + RBV 800 mg (n = 11) daily for 12 weeks. Patients receiving Dactavira or Dactavira plus had a significantly more rapid rate of viral load decline as compared to patients receiving the standard of care therapy. Sustained virological response for 12 weeks for Dactavira or Dactavira plus showed no statistically significant difference when compared to the standard of care. Also, they did not affect normal hemoglobin levels (*p* < 0.001) versus the standard of care. The incorporated EGCG interferes with the viral entry mechanisms, as reported by several investigators, and in turn enhances efficacy and prevents relapse as compared to the standard of care. Also, its antihemeolytic and antifibrotic activities may improve the safety and tolerability of the therapy.

## Introduction

Hepatitis C virus (HCV) is the common cause of liver cirrhosis and hepatocellular carcinoma^1^. It infects more than 71 million people worldwide with a high prevalence of genotype 4 virus in Egyptian patients^2^. To date, there is noavailable vaccine against HCV infection^3^.

Discovery of new molecules that act directly on the virus itself, directly acting antivirals (DAAs), which disrupt viral translation and replication by targeting viral component(s), have completely changed chronic hepatitis C (CHC) therapeutic strategies^4–7^. Most of these molecules are associated with high efficacy and safety profiles for most patients with HCV^8^. DAA combinations in all-oral regimens have led to overall high SVR (~90%) in treated individuals compared to ~60% with treatment completion of PEG IFN-α and ribavirin (RBV), independent of the severity of liver disease and viral genotype. Although DAAs have replaced IFN-based therapy, RBV remains essential in selected cases identified by the virus’ genotype, previous treatment failure, and disease stage^5–7,9,10^.

By 2013–2014, anti-HCV therapy moved a step forward after the approval of a second generation of DAAs, e.g., sofosbuvir (SOF), nucleoside/nucleotide analogues and daclatasvir (DCV), and a nonstructural protein 5A (NS5A) inhibitor^11^. SOF in combination with RBV, as well as triple therapy along with PEG IFN-α/RBV, has proven its clinical efficacy against different HCV genotypes^11,12^. The marked simplifications for such therapeutic regimens include once-daily dosing, better tolerability, improved adverse event profile, fewer drug–drug interactions, and possibly lower contraindications^13,14^.

In 2015, the combination of DCV and SOF with or without RBV as an oral treatment option for patients with chronic HCV was approved by the FDA^15^. This combination was associated with higher SVR rates, fewer adverse events, and less antiviral resistance^15^. Headache and fatigue were reported as the most common adverse effects in patients treated with the combination of SOF and DCV (~10% of patients). Furthermore, the risk of adverse effects increased in patients treated with the combination of SOF, DCV, and RBV including anemia, headache, fatigue, and nausea^16^.

In our previous study, we stated the pharmacological potential effect of EHCV (Catvira), SOF 400 mg/RBV 1000 mg/epigallocatechin gallate (EGCG) 400 mg, which showed no statistical significant difference when compared to the standard of care (SOF 400 mg/RBV 1000 mg). In that study, we reported the faster rate of viral load decline and also the protective effect of EHCV (Catvira) on red blood cell (RBC) count and hemoglobin levels as compared to the standard of care^17^. These effects could be related to the incorporation of EGCG, a polyphenol component extracted from green tea that has been shown to have multiple effects on human pathological and physiological processes including anticancer, anti-oxidant, anti-inflammatory, and antifibrosis effects^18,19^. Recently, EGCG has been identified as an inhibitor of HCV entry as well as an antihemeolytic agent^20,21^. We aimed to develop a single daily fixed-dose therapy against HCV infection that would be more effective, inexpensive, better-tolerated, and safer than the standard of care therapy. The present study compared Dactavira and Dactavira plus to the standard of care treatment: Dactavira (SOF 400 mg/DCV 60 mg/EGCG 400 mg without RBV) to standard of care (SOF 400 mg/DCV 60 mg), and Dactavira plus (includes RBV 800 mg) to the standard of care with RBV 800 mg in patients with CHC genotype 4.

## Methods

### Patients

Patients were screened and enrolled in this phase 3 open-label study at a single center in Egypt between September, 2016 and November, 2017 (ClinicalTrials.gov Identifier NCT03186313, 14/06/2017). The study was conducted in accordance with the guidelines of Good Clinical Practice and was approved by the Independent Review Board of the Faculty of Medicine, Mansoura University. All patients provided written informed consent.

Patients were required to be at least 18 years of age with body mass index ≥18 kg/m^2^ and have chronic genotype 4 HCV infection with a serum HCV RNA level ≥ 4 log_10_ IU/ml. HCV RNA was extracted from 650 µl of serum for CAP/CTM HCV v2.0 by means of the Cobas Ampliprep automated extractor, according to the manufacturer’s instructions. The Cobas Taqman 48 analyzer was used for automated real-time PCR amplification and detection of PCR products according to the manufacturer’s instructions (Roche Molecular Systems, Pleasanton, CA, USA) with a detection limit of 15 IU/ml. HCV RNA-positive samples were genotyped using an HCV real-time genotype kit (AmpliSens HCV-genotype-FRT PCR kit) that was able to detect HCV genotypes 1a, 1b, 2, 3, and 4, following the manufacturer’s instructions. The presence of cirrhosis was determined based on Fibroscan. Cirrhosis is defined by fibroscan >12·5 kpa. Laboratory test requirements included alanine aminotransferase (ALT) and aspartate aminotransferase (AST) ≤10 times the upper limit of normal (ULN), direct bilirubin ≤1·5 times ULN, hemoglobin ≥12 g/dl for men and ≥11 g/dl for women, and creatinine clearance ≥60 ml/min (Cockcroft-Gault). Consistent with the inclusion of patients with cirrhosis, patients with platelets >50,000/µl were eligible for participation. Patients with hepatitis B or HIV were excluded. Patients were treatment-naïve. Patients who had prior treatment with an anti-HCV direct-acting antiviral were excluded. Patient characteristics are compiled in Table 1.

**Table 1:** Baseline characteristics of patients.

	Median (IQR) or Frequency (%)
Age (years)	46·0 (39·0–52·0)
Gender	
- Males	41 (51·2%)
- Females	39 (48·8%)
Log_10_ PCR	5·54 (5·05–6·04)
ALT (U/L)	34·55 (26·42–49·23)
AST (U/L)	26·50 (22·00–37·50)
Total Bilirubin (mg/dl)	0·52 (0·50–0·70)
Albumin (g/dl)	4·50 (4·30–4·63)
Platelets count (/cmm^3^)	227·0 (195·0–289·0)
HgB (g/dl)	14·20 (13·10–15·40)
WBCs (/cmm^3^)	6·60 (5·03–7·62)
AFP (ng/ml)	2·72 (1·95–4·06)

### Randomization and masking

Patients were randomly assigned, on a 1:1 basis and in a masked fashion, to group A consisting of treatment-naïve patients with non-cirrhotic genotype 4 HCV infection (n = 50). They were randomly assigned in a 1:1 ratio to receive single (2 tablets) fixed-dose Dactavira daily for 12 weeks. Group B consisted of treatment-naïve patients with cirrhotic genotype 4 HCV infection (n = 22). They were randomly assigned in a 1:1 ratio to receive single (2 tablets) fixed-dose Dactavira plus daily for 12 weeks.

### Study design

Group A consisted of treatment-naïve patients with non-cirrhotic genotype 4 HCV infection (n = 50). They were randomly assigned in a 1:1 ratio to receive single (2 tablets) fixed-dose Dactavira, each tablet containing SOF 200 mg, DCV 30 mg, and EGCG 200 mg daily for 12 weeks (n = 25) or the standard of care SOF 400 mg, DCV 60 mg (n = 25) daily for 12 weeks.

Group B consisted of treatment-naïve patients with cirrhotic genotype 4 HCV infection (n = 22). They were randomly assigned in a 1:1 ratio to receive single (2 tablets) fixed-dose Dactavira plus, each tablet containing SOF 200 mg, DCV 30 mg, EGCG 200 mg, and RBV 400 mg daily for 12 weeks (n = 11) or the standard of care SOF 400 mg, DCV 60 mg with RBV 800 mg (n = 11) daily for 12 weeks. The primary efficacy endpoint was the proportion of all randomized patients who achieved SVR 12 weeks after the end of treatment (SVR12). Secondary efficacy endpoints included SVR4, on-treatment virologic failure, and virologic relapse after the end of treatment.

### Efficacy assessments

Serum HCV RNA was measured using the COBAS TaqMan HCV Test v2·0 (Roche Molecular Systems; lower limit of quantitation (LLOQ) of 25 IU/ml) at baseline, at all subsequent study visits during treatment, and post-treatment weeks 4 and 12. Patients with confirmed HCV RNA <LLOQ at the end of treatment and post-treatment visits continued to the subsequent post-treatment visits, unless confirmed virologic relapse occurred. On-treatment virologic failure was defined as: breakthrough, i.e., confirmed HCV RNA ≥LLOQ after having previously had HCV RNA <LLOQ while on-treatment; rebound, i.e., confirmed > one log IU/ml increase in HCV RNA from nadir while on-treatment; or non-response, i.e., HCV RNA persistently ≥LLOQ through 8 weeks of treatment. Relapse was defined as confirmed HCV RNA ≥LLOQ during the post-treatment period having achieved HCV RNA <LLOQ at the end of treatment.

### Safety assessments

Safety was evaluated by assessment of clinical laboratory tests, physical examination, vital sign measurements, and documentation of adverse events. Safety data were collected starting from the first dose of study medication through 30 days after the last dose.

### Statistical assessments

The primary efficacy analysis, SVR12 rates were calculated for each treatment group, along with 2-sided 95% confidence intervals (CIs) based on the Clopper-Pearson exact method. No statistical hypothesis testing was performed.

### Role of the funding source

The ELRIAH had a role in the study design, data collection, and data analysis. The corresponding author had full access to all the data in the study and had final responsibility for the decision to submit for publication.

## Results

### Patients

Eighty patients were assessed for eligibility; 6 were excluded because they did not meet the eligibility criteria. Fifty-two patients were enrolled in group A and completed treatment for 12 weeks; 26 of them received Dactavira and the other 26 received standard of care; 2 patients were lost to follow-up. Twenty-two patients were enrolled in group B and completed treatment for 12 weeks; 11 of them received Dactavira plus and the other 11 received standard of care with RBV. The mean age of enrolled patients (male and female) was 18 years, and 63% were men. All patients had a body mass index ≥18 kg/m^2^, 30·6% had cirrhosis, hemoglobin level was around >12 g/dl, and the liver enzymes (ALT and AST) were around 88·5–32·8 U/L.

### Efficacy

**Figure 1:**
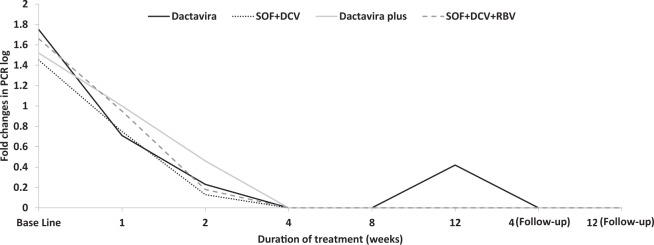
Fold change in viral load with Dactavira (SOF + DCV + EGCG) (n = 25) and Dactavira plus (SOF + DCV + EGCG + RBV) (n = 11) versus SOF + DCV (n = 25) and SOF + DCV + RBV (n = 11) in treatment-experienced patients with HCV after 12 weeks of treatment and 12 weeks post-therapy as follow-up.

Both Dactavira or Dactavira plus and the standard of care Groups A and B yielded similar treatment outcomes in terms of viral load measured at the conclusion of the 12 week study period (Fig. 1). Patients receiving Dactavira or Dactavira plus, however, had a significantly more rapid rate of viral load decline as compared to patients receiving the standard of care therapy. Patients receiving Dactavira or Dactavira plus showed a rapid decline in HCV RNA levels upon initiation of treatment, from a mean of 1·75/1·52 log_10_ at baseline to 0·0/0·42 log_10_ in the 12 weeks after treatment. On the other hand, patients receiving standard of care therapy (SOF + DCV) or (SOF + DCV + RBV) showed a decline in HCV RNA levels upon initiation of treatment, from a mean of 1·45/1·66 log_10_ at baseline to 0·0/0·0 log_10_ in the 12 weeks after treatment. Regarding the Dactavira plus group, SVR12 was achieved by all patients (100%) receiving 12 weeks of treatment (Table 2).

**Table 2:** Mean log_10_ PCR observed in patients receiving Dactavira, Dactavira plus, and standard of care with and without ribavirin (RBV), n = 72.

	Dactavira (n = 25)	SOF + DCV (n = 25)	*P* value	Dactavira plus (n = 11)	SOF + DCV + RBV (n = 11)	*P* value	*P* value All
Screening	5·93	5·64	0·074	5·76	5·8	0·921	0·404
Base Line	1·75	1·45	0·234	1·52	1·66	0·826	0·722
Week 1	0·71	0·75	0·873	1	0·95	0·915	0·856
Week 2	0·23	0·13	0·490	0·46	0·18	0·437	0·574
Week 4	0	0	NA	0	0	NA	NA
Week 8	0	0	NA	0	0	NA	NA
Week 12	0·42	0	0·132	0	0	NA	0·350
Follow-up 4 weeks	0	0	NA	0	0	NA	NA
Follow-up 12 weeks	0	0	NA	0	0	NA	NA

DCV = daclatisvir. SOF = sofosbuvir.

### Safety

In general, Dactavira or Dactavira plus related adverse effects were similar or less than in the standard of care groups, and they were observed to be less severe and persist for shorter duration. All these adverse events resolved and none was considered to be treatment-related.

**Figure 2:**
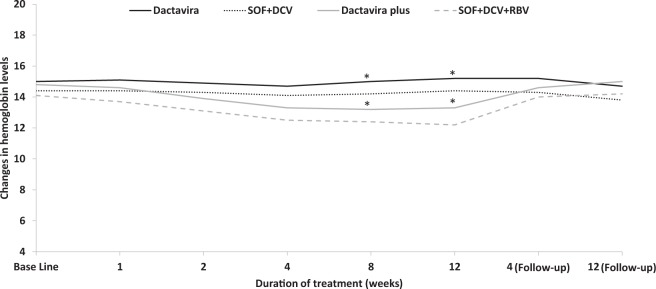
Effects on hemoglobin levels of Dactavira (SOF + DCV + EGCG) (n = 25) and Dactavira plus (SOF + DCV + EGCG + RBV) (n = 11) versus SOF + DCV (n = 25) and SOF + DCV + RBV (n = 11) in the different enrolled groups (12 weeks treatment). Values represent the changes in hemoglobin. Dactavira plus was associated with significantly less hemolysis (*p < 0·001) when compared with SOF + DCV + RBV group, and Dactavira was associated with significantly less hemolysis (*p < 0·001) when compared with SOF + DCV.

**Figure 3:**
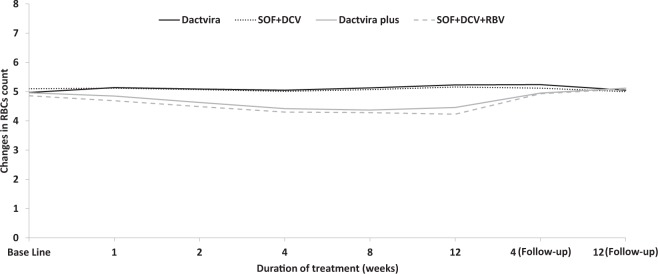
Effects on red blood cell count of Dactavira (SOF + DCV + EGCG) (n = 25) and Dactavira plus (SOF + DCV + EGCG + RBV) (n = 11) versus SOF + DCV (n = 25) and SOF + DCV + RBV (n = 11) in treatment-experienced HCV patients (12 weeks treatment). Values represent the changes in red blood cell count. Dactavira and Dactavira plus were associated with significant improvement (*p  < 0·001) when compared with SOF + DCV and SOF + DCV + RBV groups.

Consistent with changes in clinical laboratory values, Dactavira or Dactavira plus did not impact hemoglobin levels or RBC count while the standard of care resulted in a significant decline in both parameters after 12 weeks of treatment (*p* < 0·001) (Table 3, Figs 2 and 3). Neither Dactavira or Dactavira plus, nor the standard of care was associated with significant effect on platelet count (Supplementary Table S1).

**Table 3:** Mean of hemoglobin level (g/dl) observed in patients receiving Dactavira, Dactavira plus, and standard of care with and without ribavirin (RBV), n = 72.

	Dactavira	SOF + DCV	*P* value	Dactavira plus	SOF + DCV + RBV	*P* value	*P* value
	(n = 25)	(n = 25)	I vs III	(n = 11)	(n = 11)	II vs IV	All
Screening	14·9	14·3	0·109	14·6	14·1	0·421	0·294
Base Line	15·0	14·4	0·144	14·8	14·1	0·371	0·353
Week 1	15·1	14·4	0·055	14·6	13·7	0·191	0·070
Week 2	14·9	14·3	0·125	13·9	13·1	0·317	0·027
Week 4	14·7	14·1	0·102	13·3	12·5	0·205	0·002
Week 8	15·0	14·2	0·056	13·2	12·4	0·285	<0·001
Week 12	15·2	14·4	0·043	13·3	12·2	0·127	<0·001
Follow-up 4 weeks	15·2	14·3	0·014	14·6	14·0	0·385	0·047
Follow-up 12 weeks	14·7	13·8	0·058	15·0	14·2	0·241	0·126

DCV = daclatisvir. SOF = sofosbuvir.

Also, we compared the effectiveness of Dactavira, Dactavira plus, and the standard of care on liver functions including ALT, AST, bilirubin, and albumin. All treatments improved liver functions (Supplementary Table S2). Furthermore, all treatments achieved significant improvement in liver enzymes (ALT and AST) (Table 4). Regarding the diabetic status of our patients, just 6 out of 72 patients were diabetic, and we didn’t record any renal toxicity on those patients during or after the treatment.

**Table 4:** Mean of alanine aminotransferase (ALT) and aspartate aminotransferase (AST) enzymes (IU/L) observed in patients receiving Dactavira, Dactavira plus, and standard of care with and without ribavirin (RBV), n = 72.

		Dactavira (n = 25)	SOF + DCV (n = 25)	*P* value	Dactavira plus (n = 11)	SOF + DCV + RBV (n = 11)	*P* value	*P* value All
Mean of (ALT) enzyme	Screening	47·32	43·22	0·595	88·59	61·17	0·234	0.006
	Base Line	43·99	43·72	0·968	75·96	72·53	0·868	0.008
	Week 1	22·17	20·44	0·505	38·24	34·56	0·713	0.001
	Week 2	17·544	16·482	0·577	23·746	24·687	0·844	0.015
	Week 4	15·77	15·04	0·640	23·28	21·8	0·786	0·008
	Week 8	18·67	15·61	0·115	22·85	21·92	0·860	0·057
	Week 12	17·24	15·86	0·389	21·04	21·92	0·821	0·05
	Follow-up 4 weeks	29·1	16·78	0·267	21·44	25·5	0·286	0·668
	Follow-up 12 weeks	17·28	15·22	0·172	22·52	20·61	0·623	0·011
Mean of (AST) enzyme	Screening	32·86	36·33	0·392	77·46	60·23	0·446	<0·001
	Base Line	34·42	36·01	0·646	64·55	64·16	0·981	<0·001
	Week 1	20·06	20·28	0·904	31·84	27·98	0·614	0·003
	Week 2	19·04	20·15	0·390	22·85	25·21	0·584	0·045
	Week 4	18·5	19·29	0·489	24·92	23·01	0·574	0·005
	Week 8	20·63	19·88	0·651	23·35	24·73	0·716	0·206
	Week 12	19·42	20·62	0·383	22·57	25·42	0·379	0·05
	Follow-up 4 weeks	23·97	21·74	0·596	24·12	27·69	0·324	0·798
	Follow-up 12 weeks	20·37	20·03	0·814	23·44	23·38	0·987	0·284

DCV = daclatisvir. SOF = sofosbuvir.

In conclusion, there were no patients with adverse events that led to dose modification, interruption, or discontinuation of Dactavira or Dactavira plus.

## Discussion

This phase 3, open-label, 12-weeks treatment study with Dactavira or Dactavira plus resulted in high rates of SVR12 in treatment-naïve non-cirrhotic and treatment-naïve cirrhotic patients with genotype 4 HCV infection. SVR12 for Dactavira or Dactavira plus showed no statistically significant difference when compared to the standard of care. Both Dactavira and Dactavira plus were well tolerated, with mostly mild adverse events. Dactavira plus did not affect hemoglobin levels as compared to the standard of care, which resulted in a significant decline.

Dactavira or Dactavira plus may be superior to EHCV (Catvira), which was reported in our previous study to be associated with rapid decline in viral load^17^. This may be related to combination of different DAA (DCV + SOF) that act by different targeting mechanisms: NS5A inhibitor and a nucleotide NS5B inhibitor. Furthermore, as for Catvira, Dactavira plus did not show any effect on RBC count or hemoglobin levels as compared to the standard of care that resulted in a significant decline in both parameters after 12–24 weeks of treatment^17^.

Incorporation of 400 mg EGCG, which is the bio-active catechin in green tea, in Dactavira and Dactavira plus may interfere with the close links between HCV and lipid metabolism. Binding of HCV particles to hepatocytes is the intial and important step in HCV infectivity. This interaction between HCV particles and the host hepatocyte is mediated mainly by heparin sulfate proteoglycan, scavenger receptor (SR-B1), apoE, and LDL receptor (LDL-r)^22–24^.

Consistent with our results, Calland *et al*. reported that significant decrease of JFH1-Luc infection with recombinant HCV was observed in the presence of increasing concentrations of EGCG. EGCG at 5 μM was associated with more than 50% decrease in infectivity, while more than 90% decrease in infectivity was observed at 50 μM^25^.

Recent studies have reported the ability of EGCG to induce a critical structural alternation in the HCV particle itself. This structural disruption interferes with the membrane binding mediated by heparin sulfate- or sialic acid-containing glycans^20^.

Several reports have demonstrated the ability of EGCG to interfere with viral replication. An *in vitro* study using JFH1 cell line treated with 80 μM of EGCG was associated with undetecable HCV RNA and protein expression 72 hours post-infection^26^. Also, Lin *et al*. reported that HCV RNA levels were markedly eliminated (~85%) after exposure to 75 µM epicatechin isomers at 9 days using Ava5 cells^27^.

Calland *et al*. and Lin *et al*. reported the safety of EGCG with no effect on cell viability as measured with the MTS assay^25,27^. Calland *et al*. reported that EGCG had no toxic effect on Huh-7 cells (LD50 ~160 μM)^25^. Chow *et al*. concluded that EGCG is safe for healthy individuals even in content equivalent to the amount of EGCG in 16 cups of green tea (~800 mg EGCG) taken once a day or in divided doses twice a day for 4 weeks^28^.

EGCG is known as a highly efficient free radical scavenger (potent anti-oxidant). Here, we demonstrated the protective effect of Dactavira plus against hemoolytic anemia, which is associated with RBV. In our study, Dactavira plus did not affect hemoglobin levels as compared to the standard of care. Consistent with our results, Kim *et al*. reported that EGCG lowered the levels of hemolysis and hydrogen peroxide and malondialdehyde produced by normal human erythrocytes (RBCs) incubated with cyclosporine by about ~20%. This protective effect related mainly to a decrease in production of free radical species induced by the cyclosporine therapy^21^. Moses *et al*. investigated the effect of EGCG against paraquat-induced hemolysis. Paraquat is known to be toxic to RBCs’ membrane due to its redox activity, which produces superoxide anions. Osmotic fragility of the erythrocytes was tested after incubation of RBCs with different concentrations of EGCG (0·03–30 mg/ml) in addition to 30 mg/ml of paraquat for 10 minutes. EGCG (30 mg/ml) significantly (*p* < 0·05) reduced the hemolysis of erythrocytes exposed to paraquat^29^.

On the basis of these preliminary data, Dactavira and Dactavira plus, which consist of a combination of SOF, DCV, RBV, and EGCG might be superior to the standard of care and other IFN-containing regimens. Dactavira and Dactavira plus not only interfere with viral replication, but also inhibit virus entry and decrease the risk of anemia, sparing patients the rigors and toxicity of protracted IFN therapy. However, any such conclusions need to be established on a larger number of patients.

Limitations of this study include the small sample size, especially cirrhotics, and genotype subtypes were not done; results must be confirmed with larger studies. Although most cases now are treated with a RBV-free regimen because of RBV’s side effects, RBV has a crucial role in increasing SVR for treatment-expierenced and cirrhotic patients. Dactavira plus’ antihemolytic activity improves the tolerabiltity of the therapy.

In summary, 12 weeks’ therapy with the novel formulation Dactavira or Dactavira plus is safe and effective in both naïve non-cirrhotic and cirrhotic patients with genotype 4 HCV. Incorporation of EGCG in Dactavira and/Dactavira plus interferes with the viral entry mechanisms and may also play a role in enhancing efficacy and preventing relapse compared to the standard of care. In addition to potentially ehanced efficacy, Dactavira and Dactavira plus’ antihemoolytic activity may improve the safety and tolerability of the therapy.

### Compliance with ethical requirements

The study (ClinicalTrials.gov Identifier NCT03186313, 14/06/2017) was conducted in accordance with the guidelines of Good Clinical Practice and was approved by the Independent Review Board of the Faculty of Medicine, Mansoura University. All patients provided written informed consent. Special thanks to the Egyptian Liver Research Institute and Hospital (ELRIAH) clinical trial team and Virothera LLC for supporting the manufacturing and QC for Dactavira and Dactavira plus clinical batches.

## Supplementary information

Supplementary
