# Supplementary material for: Addition of Epigallocatechin Gallate 400 mg to Sofosbuvir 400 mg + Daclatisvir 60 mg With or Without Ribavirin in Treatment of Patients with Chronic Hepatitis C Improves the Safety Profile: A Pilot Study
Source: Sci Rep. 2019 Sep 19;9:13593. doi: 10.1038/s41598-019-49973-6 (PMC6753069; doi:10.1038/s41598-019-49973-6)
Supplement: Supplementary file 1 — Supplementary [file 41598_2019_49973_MOESM1_ESM.docx]

**Supplementary**

**Addition of Epigallocatechin Gallate 400 mg to Sofosbuvir 400 mg + Daclatisvir 60 mg With or Without Ribavirin in Treatment of Patients with Chronic Hepatitis C Improves the Safety Profile: A Pilot Study**

^1,2^Gamal Shiha, ^2,3^Reham Soliman, ^1,2^Mohamed Elbasiony, ^4,5^ Noureldien H. E. Darwish, ^5*^Shaker A. Mousa

***Correspondence:**

Shaker A. Mousa

The Pharmaceutical Research Institute

Albany College of Pharmacy and Health Sciences

1 Discovery Drive, Rensselaer, NY 12144

Phone: (518) 694-7397

Fax: (518) 694-7567

Email: shaker.mousa@acphs.edu

**Contents:**

**Supplementary Table S1.** Mean of platelets count (k/µL) observed in patients receiving Dactavira, Dactavira plus, and standard of care with and without ribavirin.

**Supplementary Table S2.** Mean of total bilirubin (mg/dl) observed in patients with Dactavira, Dactavira plus, and standard of care with and without ribavirin.

**Supplementary Table S3.** Mean of serum albumin (g/dl) observed in patients receiving Dactavira, Dactavira plus, and standard of care with and without ribavirin.

**Table S1.** Mean of platelets count (k/µL) observed in patients receiving Dactavira, Dactavira plus, and standard of care with and without ribavirin (RBV), n= 72

|  | Dactavira  (n=25) | SOF+DCV  (n=25) | *P* value | Dactavira plus  (n=11) | SOF+DCV+RBV  (n=11) | *P* value | *P* value  All |
| --- | --- | --- | --- | --- | --- | --- | --- |
| Screening | 196·2 | 203·1 | 0·667 | 152·4 | 166·6 | 0·624 | 0·132 |
| Base Line | 201·2 | 208·6 | 0·637 | 150·8 | 175 | 0·364 | 0·114 |
| Week 1 | 196·5 | 204·4 | 0·557 | 179 | 174·6 | 0·882 | 0·389 |
| Week 2 | 197·2 | 206·8 | 0·539 | 181·4 | 187·3 | 0·544 | 0·674 |
| Week 4 | 198·2 | 212·5 | 0·319 | 182·1 | 202·4 | 0·563 | 0·556 |
| Week 8 | 203·4 | 209·4 | 0·711 | 192·9 | 198·3 | 0·872 | 0·907 |
| Week 12 | 210·5 | 216·2 | 0·701 | 190·7 | 205·3 | 0·667 | 0·745 |
| Follow-up 4 weeks | 208·7 | 215·1 | 0·665 | 169·7 | 196·5 | 0·456 | 0·249 |
| Follow-up 12 weeks | 207·2 | 210·3 | 0·848 | 178·3 | 185 | 0·833 | 0·468 |

DCV = daclatisvir. SOF = sofosbuvir.

**Table S2.** Mean of total bilirubin (mg/dl) observed in patients with Dactavira, Dactavira plus, and standard of care with and without ribavirin (RBV), n= 72

|  | Dactavira  (n=25) | SOF+DCV  (n=25) | *P* value | Dactavira plus  (n=11) | SOF+DCV+RBV  (n=11) | *P* value | *P* value  All |
| --- | --- | --- | --- | --- | --- | --- | --- |
| Screening | 0·6 | 0·65 | 0·605 | 0·99 | 0·77 | 0·326 | 0·06 |
| Base Line | 0·58 | 0·64 | 0·412 | 0·78 | 0·7 | 0·647 | 0·471 |
| Week 1 | 0·58 | 0·68 | 0·257 | 1·17 | 1·09 | 0·810 | 0·001 |
| Week 2 | 0·63 | 0·68 | 0·586 | 1·27 | 1·06 | 0·484 | <0·001 |
| Week 4 | 0·58 | 0·64 | 0·458 | 1·02 | 1·05 | 0·925 | 0·001 |
| Week 8 | 0·54 | 0·66 | 0·240 | 0·88 | 1·09 | 0·391 | 0·006 |
| Week 12 | 0·57 | 0·57 | 0·968 | 0·88 | 0·88 | 0·987 | 0·039 |
| Follow-up 4 weeks | 0·57 | 0·61 | 0·692 | 0·56 | 0·42 | 0·238 | 0·712 |
| Follow-up 12 weeks | 0·68 | 0·61 | 0·527 | 0·72 | 0·39 | 0·053 | 0·375 |

DCV = daclatisvir. SOF = sofosbuvir.

**Table S3.** Mean of serum albumin (g/dl) observed in patients receiving Dactavira, Dactavira plus, and standard of care with and without ribavirin (RBV), n= 72

|  | Dactavira  (n=25) | SOF+DCV  (n=25) | *P* value | Dactavira plus  (n=11) | SOF+DCV+RBV  (n=11) | *P* value | *P* value  All |  |
| --- | --- | --- | --- | --- | --- | --- | --- | --- |
| Screening | 4·57 | 4·42 | 0·133 | 3·98 | 4·06 | 0·753 | 0·001 | |
| Base Line | 4·6 | 4·5 | 0·234 | 3·91 | 4·14 | 0·310 | <0·001 | |
| Week 1 | 4·66 | 4·5 | 0·036 | 4·17 | 4 | 0·443 | <0·001 | |
| Week 2 | 4·53 | 4·5 | 0·735 | 4·11 | 4·12 | 0·956 | <0·001 | |
| Week 4 | 4·53 | 4·53 | 0·975 | 4·22 | 4·26 | 0·867 | 0·028 | |
| Week 8 | 4·81 | 4·52 | 0·003 | 4·2 | 4·21 | 0·930 | <0·001 | |
| Week 12 | 4·64 | 4·45 | 0·018 | 4·19 | 4·13 | 0·720 | <0·001 | |
| Follow-up 4 weeks | 4·49 | 4·3 | 0·018 | 4·16 | 4 | 0·327 | 0·001 | |
| Follow-up 12 weeks | 4·51 | 4·36 | 0·060 | 4·26 | 4·08 | 0·376 | 0·011 | |

DCV = daclatisvir. SOF = sofosbuvir.
